# 3-(6-Methyl-2-pyrid­yl)-2-phenyl-3,4-dihydro-1,3,2-benzoxaza­phosphinine 2-oxide

**DOI:** 10.1107/S1600536809040367

**Published:** 2009-10-10

**Authors:** V. H. H. Surendra Babu, M. Krishnaiah, M. Anil Kumar, C. Suresh Reddy, Rajni Kant

**Affiliations:** a Department of Physics, S.V. University, Tirupati 517 502, India; b Department of Chemistry, S.V. University, Tirupati 517 502, India; c Department of Physics, University of Jammu, Jammu Tawi 180 006, India

## Abstract

In the title compound, C_19_H_17_N_2_O_2_P, the six-membered 1,3,2-oxaza­phosphinine ring adopts a boat conformation with the phosphoryl O atom in an equatorial position. The dihedral angle between the 6-methyl-2-pyridyl and phenyl groups is 75.5 (1)°. These substituents are *trans* to each other, and are oriented at angles of 57.2 (1) and 74.8 (1)°, respectively, to the benzene ring. The crystal structure is stabilized by intra- and inter­molecular hydrogen bonds. The phosphoryl O atom participates in inter­molecular C—H⋯O inter­actions with the neighbouring mol­ecules, forming centrosymmetric *R*_2_^2^(14) dimers.

## Related literature

For the biological activity of organophospho­rus compounds, see: Hoagland (1988 ▶); Smith (1983 ▶); Molodykh *et al.* (1990 ▶). For P—O and P=O bond lengths in related structures, see: Brzozowski *et al.* (1990 ▶); Angelov *et al.* (2002 ▶); Kant *et al.* (2009 ▶). For P—N bond lengths in related structures, see: Radha Krishna *et al.* (2007 ▶); Yang *et al.* (1988 ▶); Subramanian *et al.* (1989 ▶); Selladurai & Subramanian (1990 ▶); Selladurai *et al.* (1991 ▶).
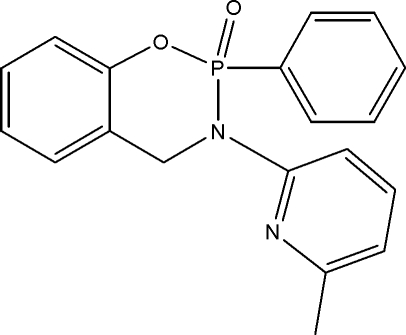


## Experimental

### 

#### Crystal data

C_19_H_17_N_2_O_2_P
*M* *_r_* = 336.32
Triclinic, 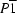

*a* = 7.2238 (8) Å
*b* = 8.6573 (8) Å
*c* = 13.7265 (14) Å
α = 95.216 (8)°
β = 94.397 (9)°
γ = 94.330 (9)°
*V* = 849.42 (15) Å^3^
*Z* = 2
Mo *K*α radiation
μ = 0.18 mm^−1^
*T* = 293 K
0.28 × 0.18 × 0.08 mm

#### Data collection

Oxford Diffraction Xcalibur diffractometer
Absorption correction: none
12457 measured reflections
5006 independent reflections
3069 reflections with *I* > 2σ(*I*)
*R*_int_ = 0.030

#### Refinement

*R*[*F* ^2^ > 2σ(*F* ^2^)] = 0.052
*wR*(*F* ^2^) = 0.173
*S* = 1.14
5006 reflections
217 parameters
H-atom parameters constrained
Δρ_max_ = 0.36 e Å^−3^
Δρ_min_ = −0.33 e Å^−3^

### 

Data collection: *CrysAlis Pro* (Oxford Diffraction, 2007 ▶); cell refinement: *CrysAlis Pro* (Oxford Diffraction, 2007 ▶); data reduction: *CrysAlis RED* (Oxford Diffraction, 2007 ▶); program(s) used to solve structure: *SHELXS97* (Sheldrick, 2008 ▶); program(s) used to refine structure: *SHELXL97* (Sheldrick, 2008 ▶); molecular graphics: *ZORTEP* (Zsolnai, 1997 ▶); software used to prepare material for publication: *enCIFer* (Allen *et al.*, 2004 ▶) and *PARST95* (Nardelli, 1995 ▶).

## Supplementary Material

Crystal structure: contains datablocks global, I. DOI: 10.1107/S1600536809040367/bh2245sup1.cif

Structure factors: contains datablocks I. DOI: 10.1107/S1600536809040367/bh2245Isup2.hkl

Additional supplementary materials:  crystallographic information; 3D view; checkCIF report

## Figures and Tables

**Table 1:** Hydrogen-bond geometry (Å, °)

*D*—H⋯*A*	*D*—H	H⋯*A*	*D*⋯*A*	*D*—H⋯*A*
C17—H17⋯O5^i^	0.93	2.57	3.455 (3)	159
C21—H21⋯O5^ii^	0.93	2.56	3.445 (3)	159
C18—H18⋯O5	0.93	2.50	3.158 (3)	128

Symmetry codes: (i) 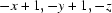; (ii) 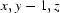.

**Fig. 1.**
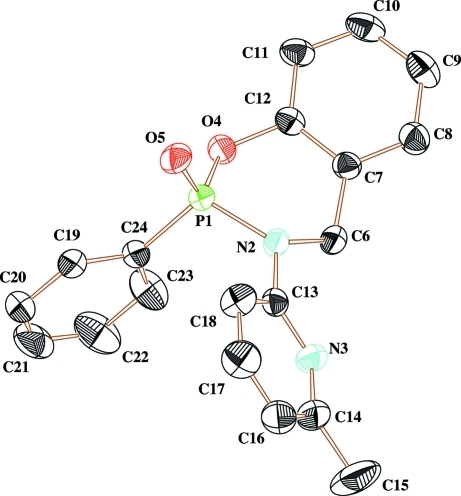
View of the molecule showing the atom-labeling scheme. Displacement ellipsoids are drawn at the 40% probability level.

**Fig. 2.**
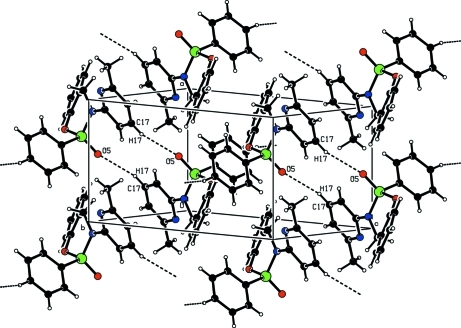
Packing of the molecules in the unit cell.

## supplementary crystallographic information

### Comment

As organophosphorus compounds are ubiquitous, they have found multifaceted applications in nature. They can be used as insecticides and herbicides (Hoagland *et al.*, 1988), fungicides (Smith *et al.*, 1983), plant growth regulators and present also antifungal activity (Molodykh *et al.*, 1990). The significant activity of all these compounds was accredited to the presence of six membered heterocyclic rings. In view of these activities, the title compound, (I), has been studied, as a part of our ongoing investigation to find out the influence of different substituents on the conformation of the heterocyclic ring.

In the molecular structure (Fig. 1), the oxazaphosphinine ring exhibits a boat conformation where atoms C6/C7/C12/O4 are almost coplanar and atoms P1 and N2 displaced in the same direction by 0.936 (1) and 0.936 (1) Å, respectively. The single and double bond lengths of P and O atoms are in good agreement with the similar structures reported previously (Brzozowski *et al.*, 1990; Angelov *et al.*, 2002; Kant *et al.*, 2009). The P—N bond length, 1.6702 (14) Å, and the P—N—C bond angle, 120.30 (13)°, are comparable with the related structure of [1,3,2]-oxazaphosphorine-6-sulfide (Radha Krishna *et al.*, 2007), but the bond distance shows relatively higher value when compared with the similar benzoxazaphosphorine structures (Yang *et al.*, 1988; Subramanian *et al.*, 1989; Selladurai & Subramanian, 1990; Selladurai *et al.*, 1991). The N3—C13 and N3—C14 bond lengths fall between the expected single bond and double bond distances, showing the partial double bond character at N3.

In the crystal structure, the phosphoryl O atom participates in intermolecular C—H···O interactions with the neighboring molecules, to form centrosymmetric *R*_2_^2^(14) dimers, along [011] (Fig. 2).

### Experimental

A solution of phenylphosphonic dichloride (0.002 mol) in 25 ml of dry THF was added dropwise over a period of 20 min. to a stirred solution of 2-{[(6-methyl-2-pyridyl)amino]methyl}phenol (0.002 mol) and triethylamine (0.004 mol) in 30 ml of dry THF. After completion of the addition, the temperature of the reaction mixture was slowly raised to room temperature and stirred for 30 min. The reaction mixture was then heated to 318–323 K and maintained at that temperature for 3 h. under stirring. Completion of the reaction was monitored by TLC analysis. Triethylamine hydrochloride was filtered from the reaction mixture and the solvent was removed under reduced pressure. The crude product was purified by column chromatography on silica gel (100–200 mesh, ethyl acetate:hexane) to afford pure product. Transparent, colorless plate-shaped single crystals are obtained by slow evaporation of a 2-proponal solution.

### Refinement

All C-bonded H-atoms were positioned geometrically and refined using a riding model with d(C—H) = 0.93 Å, *U*_iso_(H) = 1.2*U*_eq_(C) for aromatic, 0.97 Å, *U*_iso_(H) = 1.2*U*_eq_(C) for CH_2_ group and 0.96 Å, *U*_iso_(H) = 1.5*U*_eq_(C) for CH_3_ group.

### Crystal data

**Table:**

C_19_H_17_N_2_O_2_P	*Z* = 2
*M**_r_* = 336.32	*F*(000) = 352
Triclinic, *P*1	*D*_x_ = 1.315 Mg m^−^^3^
Hall symbol: -P 1	Mo *K*α radiation, λ = 0.71073 Å
*a* = 7.2238 (8) Å	Cell parameters from 5006 reflections
*b* = 8.6573 (8) Å	θ = 3.3–30.1°
*c* = 13.7265 (14) Å	µ = 0.18 mm^−^^1^
α = 95.216 (8)°	*T* = 293 K
β = 94.397 (9)°	Plate, colourless
γ = 94.330 (9)°	0.28 × 0.18 × 0.08 mm
*V* = 849.42 (15) Å^3^	

### Data collection

**Table:**

Oxford Diffraction Xcalibur diffractometer	3069 reflections with *I* > 2σ(*I*)
Radiation source: fine-focus sealed tube	*R*_int_ = 0.030
graphite	θ_max_ = 30.1°, θ_min_ = 3.3°
ω–2θ scans	*h* = −10→10
12457 measured reflections	*k* = −12→12
5006 independent reflections	*l* = −19→19

### Refinement

**Table:**

Refinement on *F*^2^	Primary atom site location: structure-invariant direct methods
Least-squares matrix: full	Secondary atom site location: difference Fourier map
*R*[*F*^2^ > 2σ(*F*^2^)] = 0.052	Hydrogen site location: inferred from neighbouring sites
*wR*(*F*^2^) = 0.173	H-atom parameters constrained
*S* = 1.14	*w* = 1/[σ^2^(*F*_o_^2^) + (0.0909*P*)^2^] where *P* = (*F*_o_^2^ + 2*F*_c_^2^)/3
5006 reflections	(Δ/σ)_max_ < 0.001
217 parameters	Δρ_max_ = 0.36 e Å^−^^3^
0 restraints	Δρ_min_ = −0.33 e Å^−^^3^
0 constraints	

### Special details

**Table:**

Refinement. Weighted least-squares planes through the starred atoms (Nardelli, Musatti, Domiano & Andreetti Ric.Sci.(1965),15(II—A),807). Equation of the plane: m1**X*+m2*Y+m3**Z*=dPlane 1 m1 = 0.38775(0.00078) m2 = 0.69013(0.00101) m3 = -0.61104(0.00092) D = -0.04785(0.00813) Atom d s d/s (d/s)**2 C6 * 0.0033 0.0020 1.652 2.729 C7 * -0.0061 0.0019 - 3.190 10.179 C12 * 0.0060 0.0019 3.207 10.286 O4 * -0.0017 0.0014 - 1.201 1.443 P1 0.9362 0.0005 1909.984 3648038.000 N2 0.9357 0.0015 617.499 381304.969 ============ Sum((d/s)**2) for starred atoms 24.637 Chi-squared at 95% for 1 degrees of freedom: 3.84 The group of atoms deviates significantly from planarityPlane 2 m1 = 0.36501(0.00090) m2 = 0.72088(0.00066) m3 = -0.58915(0.00074) D = 0.19711(0.00721) Atom d s d/s (d/s)**2 C7 * -0.0010 0.0019 - 0.547 0.300 C8 * -0.0022 0.0022 - 1.022 1.044 C9 * 0.0076 0.0024 3.196 10.214 C10 * -0.0077 0.0023 - 3.346 11.194 C11 * 0.0029 0.0021 1.390 1.931 C12 * 0.0007 0.0019 0.353 0.125 ============ Sum((d/s)**2) for starred atoms 24.808 Chi-squared at 95% for 3 degrees of freedom: 7.81 The group of atoms deviates significantly from planarityPlane 3 m1 = -0.45957(0.00079) m2 = 0.86478(0.00046) m3 = -0.20235(0.00092) D = 2.02662(0.00192) Atom d s d/s (d/s)**2 C13 * 0.0050 0.0018 2.731 7.457 N3 * -0.0045 0.0018 - 2.513 6.313 C14 * 0.0033 0.0025 1.334 1.780 C16 * 0.0013 0.0026 0.508 0.258 C17 * -0.0009 0.0027 - 0.328 0.108 C18 * -0.0040 0.0024 - 1.686 2.842 ============ Sum((d/s)**2) for starred atoms 18.758 Chi-squared at 95% for 3 degrees of freedom: 7.81 The group of atoms deviates significantly from planarityPlane 4 m1 = -0.44454(0.00100) m2 = -0.15288(0.00101) m3 = -0.88262(0.00050) D = -4.64491(0.00165) Atom d s d/s (d/s)**2 C19 * 0.0128 0.0022 5.712 32.623 C20 * -0.0095 0.0029 - 3.274 10.717 C21 * -0.0074 0.0031 - 2.395 5.738 C22 * 0.0121 0.0031 3.908 15.276 C23 * 0.0012 0.0025 0.480 0.231 C24 * -0.0084 0.0020 - 4.200 17.636 ============ Sum((d/s)**2) for starred atoms 82.221 Chi-squared at 95% for 3 degrees of freedom: 7.81 The group of atoms deviates significantly from planarityDihedral angles formed by LSQ-planes Plane - plane angle (s.u.) angle (s.u.) 1 2 2.52 (0.07) 177.48 (0.07) 1 3 57.16 (0.06) 122.84 (0.06) 1 4 74.84 (0.08) 105.16 (0.08) 2 3 54.91 (0.06) 125.09 (0.06) 2 4 75.67 (0.07) 104.33 (0.07) 3 4 75.48 (0.08) 104.52 (0.08)

### Fractional atomic coordinates and isotropic or equivalent isotropic displacement parameters (Å^2^)

**Table:**

	*x*	*y*	*z*	*U*_iso_*/*U*_eq_	
P1	0.37783 (6)	0.42101 (5)	0.26648 (4)	0.03578 (17)	
O4	0.34114 (19)	0.46439 (16)	0.37866 (10)	0.0459 (4)	
N2	0.16623 (19)	0.42897 (18)	0.20981 (11)	0.0360 (4)	
O5	0.52630 (17)	0.51923 (15)	0.23013 (11)	0.0491 (4)	
C24	0.4304 (3)	0.2230 (2)	0.26798 (15)	0.0399 (4)	
C7	0.0443 (2)	0.5645 (2)	0.35210 (14)	0.0380 (4)	
C6	0.0029 (2)	0.4431 (2)	0.26737 (15)	0.0413 (4)	
H6A	−0.0316	0.3438	0.2913	0.050*	
H6B	−0.1017	0.4703	0.2255	0.050*	
N3	−0.0324 (2)	0.2849 (2)	0.08712 (13)	0.0476 (4)	
C12	0.2155 (3)	0.5737 (2)	0.40604 (14)	0.0374 (4)	
C13	0.1314 (2)	0.3663 (2)	0.10995 (14)	0.0367 (4)	
C11	0.2642 (3)	0.6790 (2)	0.48673 (15)	0.0475 (5)	
H11	0.3809	0.6826	0.5210	0.057*	
C8	−0.0832 (3)	0.6682 (3)	0.38261 (16)	0.0485 (5)	
H8	−0.1999	0.6649	0.3484	0.058*	
C9	−0.0374 (4)	0.7763 (3)	0.46352 (17)	0.0574 (6)	
H9	−0.1226	0.8463	0.4824	0.069*	
C10	0.1326 (4)	0.7804 (3)	0.51557 (16)	0.0560 (6)	
H10	0.1607	0.8513	0.5707	0.067*	
C19	0.5868 (3)	0.1738 (2)	0.22629 (17)	0.0509 (5)	
H19	0.6631	0.2428	0.1959	0.061*	
C14	−0.0738 (3)	0.2269 (3)	−0.00667 (18)	0.0579 (6)	
C18	0.2619 (3)	0.3908 (3)	0.04171 (16)	0.0532 (5)	
H18	0.3761	0.4468	0.0603	0.064*	
C23	0.3141 (4)	0.1170 (3)	0.30964 (18)	0.0623 (7)	
H23	0.2076	0.1487	0.3370	0.075*	
C20	0.6295 (4)	0.0186 (3)	0.2303 (2)	0.0683 (8)	
H20	0.7366	−0.0145	0.2044	0.082*	
C16	0.0481 (3)	0.2471 (3)	−0.07839 (17)	0.0590 (6)	
H16	0.0157	0.2050	−0.1427	0.071*	
C21	0.5141 (5)	−0.0834 (3)	0.2720 (2)	0.0788 (9)	
H21	0.5424	−0.1861	0.2743	0.095*	
C17	0.2164 (4)	0.3297 (3)	−0.05363 (18)	0.0643 (6)	
H17	0.2996	0.3444	−0.1011	0.077*	
C22	0.3571 (5)	−0.0350 (3)	0.3103 (2)	0.0821 (9)	
H22	0.2778	−0.1060	0.3373	0.099*	
C15	−0.2625 (4)	0.1350 (5)	−0.0295 (2)	0.1035 (13)	
H15A	−0.3264	0.1337	0.0292	0.155*	
H15B	−0.2449	0.0302	−0.0542	0.155*	
H15C	−0.3352	0.1830	−0.0780	0.155*	

### Atomic displacement parameters (Å^2^)

**Table:**

	*U*^11^	*U*^22^	*U*^33^	*U*^12^	*U*^13^	*U*^23^
P1	0.0327 (3)	0.0325 (3)	0.0410 (3)	0.00243 (18)	−0.00086 (19)	0.00089 (19)
O4	0.0493 (8)	0.0471 (8)	0.0395 (8)	0.0155 (6)	−0.0083 (6)	−0.0055 (6)
N2	0.0304 (7)	0.0421 (8)	0.0352 (9)	0.0048 (6)	0.0018 (6)	0.0017 (6)
O5	0.0364 (7)	0.0426 (8)	0.0666 (10)	−0.0066 (6)	0.0011 (6)	0.0077 (7)
C24	0.0403 (9)	0.0348 (9)	0.0436 (11)	0.0058 (8)	−0.0026 (8)	0.0014 (8)
C7	0.0374 (9)	0.0386 (10)	0.0375 (10)	0.0015 (7)	0.0071 (8)	−0.0011 (8)
C6	0.0314 (9)	0.0468 (11)	0.0443 (11)	0.0041 (8)	0.0008 (8)	−0.0013 (8)
N3	0.0411 (9)	0.0550 (10)	0.0431 (10)	0.0014 (8)	−0.0024 (7)	−0.0079 (8)
C12	0.0463 (10)	0.0331 (9)	0.0334 (10)	0.0046 (8)	0.0040 (8)	0.0050 (7)
C13	0.0382 (9)	0.0363 (9)	0.0359 (10)	0.0084 (7)	0.0000 (8)	0.0026 (7)
C11	0.0627 (13)	0.0414 (10)	0.0362 (11)	0.0036 (9)	−0.0041 (9)	−0.0004 (8)
C8	0.0484 (11)	0.0517 (12)	0.0457 (12)	0.0094 (9)	0.0072 (9)	−0.0001 (9)
C9	0.0735 (15)	0.0510 (12)	0.0512 (14)	0.0206 (11)	0.0170 (12)	0.0010 (10)
C10	0.0829 (16)	0.0445 (12)	0.0380 (12)	0.0049 (11)	0.0030 (11)	−0.0075 (9)
C19	0.0358 (10)	0.0487 (12)	0.0645 (15)	0.0038 (9)	−0.0003 (9)	−0.0105 (10)
C14	0.0526 (12)	0.0661 (15)	0.0498 (14)	0.0071 (11)	−0.0090 (10)	−0.0133 (11)
C18	0.0495 (12)	0.0642 (14)	0.0446 (13)	−0.0043 (10)	0.0045 (10)	0.0051 (10)
C23	0.0888 (18)	0.0445 (12)	0.0616 (16)	0.0143 (11)	0.0293 (13)	0.0215 (10)
C20	0.0558 (14)	0.0600 (15)	0.083 (2)	0.0210 (12)	−0.0106 (13)	−0.0255 (14)
C16	0.0659 (14)	0.0711 (16)	0.0373 (12)	0.0153 (12)	−0.0047 (10)	−0.0099 (11)
C21	0.115 (2)	0.0425 (13)	0.076 (2)	0.0263 (15)	−0.0259 (17)	0.0016 (13)
C17	0.0723 (16)	0.0806 (17)	0.0402 (13)	0.0067 (13)	0.0116 (11)	0.0021 (12)
C22	0.130 (3)	0.0433 (13)	0.079 (2)	0.0139 (16)	0.0199 (19)	0.0209 (13)
C15	0.0665 (17)	0.157 (3)	0.070 (2)	−0.0246 (19)	−0.0090 (15)	−0.045 (2)

### Geometric parameters (Å, °)

**Table:**

P1—O5	1.4641 (14)	C9—C10	1.369 (3)
P1—O4	1.5984 (15)	C9—H9	0.9300
P1—N2	1.6702 (14)	C10—H10	0.9300
P1—C24	1.7850 (19)	C19—C20	1.406 (3)
O4—C12	1.407 (2)	C19—H19	0.9300
N2—C13	1.424 (2)	C14—C16	1.384 (4)
N2—C6	1.476 (2)	C14—C15	1.522 (3)
C24—C19	1.383 (3)	C18—C17	1.372 (3)
C24—C23	1.388 (3)	C18—H18	0.9300
C7—C12	1.385 (3)	C23—C22	1.376 (3)
C7—C8	1.395 (3)	C23—H23	0.9300
C7—C6	1.491 (3)	C20—C21	1.362 (4)
C6—H6A	0.9700	C20—H20	0.9300
C6—H6B	0.9700	C16—C17	1.366 (3)
N3—C13	1.331 (2)	C16—H16	0.9300
N3—C14	1.342 (3)	C21—C22	1.364 (4)
C12—C11	1.374 (3)	C21—H21	0.9300
C13—C18	1.397 (3)	C17—H17	0.9300
C11—C10	1.397 (3)	C22—H22	0.9300
C11—H11	0.9300	C15—H15A	0.9600
C8—C9	1.388 (3)	C15—H15B	0.9600
C8—H8	0.9300	C15—H15C	0.9600
			
O5—P1—O4	114.90 (8)	C8—C9—H9	119.9
O5—P1—N2	114.95 (8)	C9—C10—C11	120.7 (2)
O4—P1—N2	101.68 (7)	C9—C10—H10	119.7
O5—P1—C24	112.80 (9)	C11—C10—H10	119.7
O4—P1—C24	101.38 (8)	C24—C19—C20	119.4 (2)
N2—P1—C24	109.81 (8)	C24—C19—H19	120.3
C12—O4—P1	121.93 (12)	C20—C19—H19	120.3
C13—N2—C6	116.96 (14)	N3—C14—C16	122.4 (2)
C13—N2—P1	118.81 (12)	N3—C14—C15	115.9 (2)
C6—N2—P1	120.30 (13)	C16—C14—C15	121.7 (2)
C19—C24—C23	119.55 (19)	C17—C18—C13	117.9 (2)
C19—C24—P1	119.92 (16)	C17—C18—H18	121.0
C23—C24—P1	120.53 (16)	C13—C18—H18	121.0
C12—C7—C8	117.29 (17)	C22—C23—C24	119.7 (2)
C12—C7—C6	119.26 (16)	C22—C23—H23	120.2
C8—C7—C6	123.43 (17)	C24—C23—H23	120.2
N2—C6—C7	110.86 (15)	C21—C20—C19	120.2 (2)
N2—C6—H6A	109.5	C21—C20—H20	119.9
C7—C6—H6A	109.5	C19—C20—H20	119.9
N2—C6—H6B	109.5	C17—C16—C14	119.3 (2)
C7—C6—H6B	109.5	C17—C16—H16	120.4
H6A—C6—H6B	108.1	C14—C16—H16	120.4
C13—N3—C14	117.66 (19)	C20—C21—C22	120.0 (2)
C11—C12—C7	123.37 (18)	C20—C21—H21	120.0
C11—C12—O4	119.00 (17)	C22—C21—H21	120.0
C7—C12—O4	117.56 (16)	C16—C17—C18	119.5 (2)
N3—C13—C18	123.22 (18)	C16—C17—H17	120.2
N3—C13—N2	115.42 (16)	C18—C17—H17	120.2
C18—C13—N2	121.35 (17)	C21—C22—C23	121.2 (3)
C12—C11—C10	117.8 (2)	C21—C22—H22	119.4
C12—C11—H11	121.1	C23—C22—H22	119.4
C10—C11—H11	121.1	C14—C15—H15A	109.5
C9—C8—C7	120.6 (2)	C14—C15—H15B	109.5
C9—C8—H8	119.7	H15A—C15—H15B	109.5
C7—C8—H8	119.7	C14—C15—H15C	109.5
C10—C9—C8	120.3 (2)	H15A—C15—H15C	109.5
C10—C9—H9	119.9	H15B—C15—H15C	109.5
			
O5—P1—O4—C12	88.83 (15)	C6—N2—C13—N3	−19.5 (2)
N2—P1—O4—C12	−35.98 (15)	P1—N2—C13—N3	138.32 (15)
C24—P1—O4—C12	−149.22 (14)	C6—N2—C13—C18	160.12 (19)
O5—P1—N2—C13	67.98 (16)	P1—N2—C13—C18	−42.1 (2)
O4—P1—N2—C13	−167.24 (13)	C7—C12—C11—C10	0.5 (3)
C24—P1—N2—C13	−60.47 (16)	O4—C12—C11—C10	−176.41 (18)
O5—P1—N2—C6	−134.97 (14)	C12—C7—C8—C9	0.4 (3)
O4—P1—N2—C6	−10.20 (16)	C6—C7—C8—C9	178.5 (2)
C24—P1—N2—C6	96.57 (15)	C7—C8—C9—C10	−1.3 (4)
O5—P1—C24—C19	−7.5 (2)	C8—C9—C10—C11	1.7 (4)
O4—P1—C24—C19	−130.88 (17)	C12—C11—C10—C9	−1.3 (3)
N2—P1—C24—C19	122.15 (17)	C23—C24—C19—C20	−2.5 (3)
O5—P1—C24—C23	173.33 (17)	P1—C24—C19—C20	178.29 (17)
O4—P1—C24—C23	49.93 (19)	C13—N3—C14—C16	0.9 (3)
N2—P1—C24—C23	−57.0 (2)	C13—N3—C14—C15	−179.9 (2)
C13—N2—C6—C7	−156.56 (16)	N3—C13—C18—C17	1.1 (3)
P1—N2—C6—C7	46.0 (2)	N2—C13—C18—C17	−178.50 (19)
C12—C7—C6—N2	−41.6 (3)	C19—C24—C23—C22	0.9 (4)
C8—C7—C6—N2	140.36 (19)	P1—C24—C23—C22	−179.9 (2)
C8—C7—C12—C11	−0.1 (3)	C24—C19—C20—C21	2.2 (4)
C6—C7—C12—C11	−178.27 (19)	N3—C14—C16—C17	−0.4 (4)
C8—C7—C12—O4	176.88 (18)	C15—C14—C16—C17	−179.5 (3)
C6—C7—C12—O4	−1.3 (3)	C19—C20—C21—C22	−0.2 (4)
P1—O4—C12—C11	−138.17 (16)	C14—C16—C17—C18	0.1 (4)
P1—O4—C12—C7	44.7 (2)	C13—C18—C17—C16	−0.4 (4)
C14—N3—C13—C18	−1.3 (3)	C20—C21—C22—C23	−1.4 (4)
C14—N3—C13—N2	178.29 (17)	C24—C23—C22—C21	1.0 (4)

### Hydrogen-bond geometry (Å, °)

**Table:**

*D*—H···*A*	*D*—H	H···*A*	*D*···*A*	*D*—H···*A*
C17—H17···O5^i^	0.93	2.57	3.455 (3)	159
C21—H21···O5^ii^	0.93	2.56	3.445 (3)	159
C18—H18···O5	0.93	2.50	3.158 (3)	128

Symmetry codes: (i) −*x*+1, −*y*+1, −*z*; (ii) *x*, *y*−1, *z*.
